# Spreading the news: on the publication of scientific findings

**DOI:** 10.1590/S1980-57642009DN30400001

**Published:** 2009

**Authors:** Ricardo Nitrini

**Affiliations:** Editor-in-Chief

From the frightening “publish or perish” to the stimulating “publish and flourish”, the advice remains the same: an academic career depends on writing up the results of research and making them public. Where to publish also hinges on the idea of broadcasting the findings, and it is generally agreed that manuscripts should be first submitted to the most renowned periodicals. There are many reasons for this consensus, among them the likelihood of the manuscript being revised by important researchers in the field and, if accepted, the receipt of a kind of seal of quality. Last but not least, to be published by an important periodical increases the chances of being cited because these are read by the most productive researchers. Subsequent citation in other papers is the end goal and a measure of the quality of the contribution. This is the universal path in the search for excellence in academic life. Often times an outstanding publication paves the way for more in-depth cooperative research and has a decisive impact on academic careers.

However, manuscripts frequently present findings that are important only to a specific region or to speakers of a particular language. Moreover, papers may be more relevant to certain cultural groups than others thereby reducing their chances of publication in a prominent periodical. In the past, most of these manuscripts ended up being published in obscure local journals, often not in English. In this sense, they were never really made public. One of the worst upshots of the ostracism of these papers was the realization that sometimes the same type of research was later conducted by other investigators, who, unaware of the previous papers, repeated the same methodological errors criticized by these earlier papers. One of the main reasons for launching **Dementia & Neuropsychologia** was to bring these papers to light and to make such occurrences a thing of the past. Since the advent of electronic online publication of scientific papers, it has become easier to make results public and to retrieve papers published almost anywhere in the world.

It is now fitting to inform our authors and readers on the exposure of papers published by our journal. Are they really reaching the scientific community? Members of the societies for which **Dementia & Neuropsychologia** is the official journal receive a printed copy of each issue, but what about the scientific community elsewhere? **Dementia & Neuropsychologia** is accessible online at www.demneuropsy.com.br, where papers are freely available, and the data on those accessing the site and downloading papers may provide a clue to the visibility of the journal.

Since December 2008, when only 136 downloads of manuscripts were registered, there has been a steady rise in number of visits with downloads reaching 2,315 in October 2009, giving a grand total of 11,022 in the past 11 months. The visitors were from 66 countries, with the most frequent countries of origin of the visitors being, in descending order of number of visits: Brazil, the United States, China, Portugal, the United Kingdom, Australia, the Netherlands, Italy, Germany, Belgium, Japan, the Russian Federation, Argentina and India.

These numbers are truly rewarding for our authors and may serve as an incentive for former and future authors to submit their papers to **Dementia & Neuropsychologia.**

**Ricardo Nitrini** Editor-in-Chief

